# Intravenous Administration of Human Umbilical Cord Mesenchymal Stromal Cells Leads to an Inflammatory Response in the Lung

**DOI:** 10.1155/2023/7397819

**Published:** 2023-09-05

**Authors:** Alejandra Hernandez Pichardo, Bettina Wilm, Neill J. Liptrott, Patricia Murray

**Affiliations:** ^1^Department of Molecular Physiology and Cell Signalling, Institute of Systems, Molecular and Integrative Biology, University of Liverpool, Liverpool, UK; ^2^Centre for Pre-Clinical Imaging, Faculty of Health and Life Sciences, University of Liverpool, Liverpool, UK; ^3^Immunocompatibility Group, Department of Pharmacology and Therapeutics, Institute of Systems, Molecular and Integrative Biology, University of Liverpool, Liverpool, UK

## Abstract

Mesenchymal stromal cells (MSCs) administered intravenously (IV) have shown efficacy in preclinical models of various diseases. This is despite the cells not reaching the site of injury due to entrapment in the lungs. The immunomodulatory properties of MSCs are thought to underlie their therapeutic effects, irrespective of whether they are sourced from bone marrow, adipose tissue, or umbilical cord. To better understand how MSCs affect innate immune cell populations in the lung, we evaluated the distribution and phenotype of neutrophils, monocytes, and macrophages by flow cytometry and histological analyses after delivering human umbilical cord-derived MSCs (hUC-MSCs) IV into immunocompetent mice. After 2 hr, we observed a significant increase in neutrophils, and proinflammatory monocytes and macrophages. Moreover, these immune cells localized in close proximity to the MSCs, suggesting an active role in their clearance. By 24 hr, we detected an increase in anti-inflammatory monocytes and macrophages. These results suggest that the IV injection of hUC-MSCs leads to an initial inflammatory phase in the lung shortly after injection, followed by a resolution phase 24 hr later.

## 1. Introduction

After intravenous (IV) injection, most nonhematological cells such as mesenchymal stromal cells (MSCs) remain trapped within the lung vasculature and die within 24 hr [1, 2]. This is referred to as the first-pass effect [3] and contradicts earlier reports that suggest MSCs migrate to sites of injury and differentiate into tissue-specific cells [4]. Evidence suggests that the therapeutic benefits of MSCs are likely mediated, at least in part, by the release of trophic factors and their ability to modulate the immune system [5–7].

Several studies suggest that exogenous MSCs can ameliorate injury in a variety of animal models such as the heart [8], eye [9], kidney [10], bone [11], cartilage [12], and liver [13]. The underlying mechanisms are not fully elucidated and understanding the initial effect that MSCs have on immune cell populations in the lung after IV delivery could shed light on this question.

The first type of immune cells that respond to invading pathogens or foreign materials are the cells of the innate immune system [14]. Bone marrow-derived myeloid cells consist of a heterogeneous population that includes monocytes, macrophages, and dendritic cells (DCs), as well as granulocytes (mast cells, basophils, eosinophils, and neutrophils) [15]. Despite each subset having specialized functions based on their environment, all myeloid cells play a role in the phagocytosis of foreign materials, opsonized extracellular microbes, and dying/dead cells [16–18]. Moreover, they secrete cytokines and chemokines that regulate the immune response [19].

Neutrophils, the most abundant type of granulocytes, are the first cells recruited to sites of injury, followed by monocytes and macrophages [20, 21]. Neutrophils are known for their microbe-clearing mechanisms involving the generation of reactive oxygen species, antimicrobial protein degranulation, and the formation of neutrophil extracellular traps (NETs) [22, 23]. MSCs have been shown to mediate their therapeutic benefits by modulating neutrophils; for instance, bacterial clearance was enhanced in a murine sepsis model after IV administration of MSCs because the MSCs enhanced the phagocytic capacity of the neutrophils [24].

Monocytes are precursor cells that give rise to DCs and macrophages. Their mobility gives them a unique role in the mononuclear phagocyte system. In contrast to the limited migration potential of terminally differentiated DCs and macrophages, monocytes are rapidly mobilized upon challenge and can access any location within the body [25]. In the context of cell therapies, an IV injection of human umbilical cord-derived MSCs (hUC-MSCs) into mice showed that monocytes mediate the rapid clearance of the cells by phagocytosis [26]. Moreover, phagocytosis of the administered cells in the lung resulted in the monocytes being reprogrammed toward an anti-inflammatory activation state [26].

Tissue macrophages play various homeostatic roles such as tissue remodeling and repair, clearing of senescent cells, as well as induction and resolution of the inflammatory response [25]. In addition, macrophages engage with T and B lymphocytes and participate in the induction of adaptive immunity [27]. The IV infusion of MSCs in mice leads to an inflammatory response accompanied by increased numbers of macrophages in the lungs [28]. Moreover, 1 week after MSC administration, one study showed that the number of anti-inflammatory (M2) macrophages increased, whereas there was a decrease in inflammatory (M1) macrophages [29]. The ability of MSCs to increase the number of M2 macrophages is thought to underlie their therapeutic effects in various animal models of disease [30]. For instance, MSCs alleviated tissue damage and inflammation in a mouse model of acute kidney injury by inducing macrophage polarization toward an anti-inflammatory M2 phenotype within 24 hr following IV administration of the MSCs [31].

Macrophages can further be categorized based on their location. In the lungs, resident alveolar macrophages are maintained by local proliferation [32] and perform tissue-specific roles such as surfactant clearance [33]. MSCs reduced the severity of lung injury in an *Escherichia coli* pneumonia model and modulated alveolar macrophage polarization in vivo [34]. Interstitial macrophages mature in the lungs after the recruitment of precursors from the blood [25]. Their localization within the lungs remains unclear, but studies have shown their presence in the parenchyma [35] and the bronchial interstitium [36]. The functions of lung-resident macrophages include phagocytosis of foreign invaders, antigen presentation, and immune modulation [37].

Some studies have investigated the interactions between infused MSCs and specific immune cell populations in vivo [26, 38, 39], but a comprehensive analysis on the impact of innate cells is lacking, especially in the period immediately following MSC administration. In this study, we have used hUC-MSCs because of reports suggesting that they have superior immunomodulatory properties compared to those derived from bone marrow [40]. Our aim was to investigate the fate of hUC-MSCs in the lungs and their effect on the proportion, distribution, and polarization state of innate immune cell populations, particularly granulocytes, monocytes, and macrophages. To do this, we administered cells via the tail vein into immunocompetent naïve mice. Then, we measured the changes in myeloid cells at the following time points: 2 hr (when most hUC-MSCs are still viable) and 24 hr (when most hUC-MSCs had been cleared).

## 2. Methods

### 2.1. Cell Preparation

Primary hUC-MSCs were collected from consenting donors by the National Health Service Blood and Transplant (NHSBT, UK) and transferred to the University of Liverpool at passage 3. In this study, experiments were conducted with cells from a single donor.

hUC-MSCs were transduced in the presence of 6 *µ*g/ml DEAE-Dextran with a lentiviral vector pCDH-EF1-Luc2-P2A-tdTomato, encoding luc2 firefly luciferase (FLuc) reporter under the constitutive elongation factor 1-*α* (EF1*α*) promoter and upstream of a P2A linker followed by the tdTomato fluorescent protein (gift from Kazuhiro Oka; Addgene plasmid # 72486; http://n2t.net/addgene: 72486; RRID: Addgene_72486). To obtain a >98% transduced population, the cells were sorted based on tdTomato fluorescence (BD FACS Aria). The cells were cultured in *α*-MEM supplemented with 10% FBS and incubated at 37°C, 5% CO_2_.

### 2.2. Animal Studies

All experiments were carried out under a license granted under the UK Animals Act 1986 and approved by the ethics committee of the University of Liverpool Animal Welfare and Ethics Review Board. Eight- to ten-week-old female albino mice (C57BL/6) (B6N-TyrC-Brd/BrdCrCrl, originally purchased from the Jackson Lab) were housed in individually ventilated cages under a 12 hr light/dark cycle, with ad libitum access to water and food.

### 2.3. Dissociation of Lung Tissue

hUC-MSCs were suspended in ice–cold phosphate-buffered saline (PBS) and were kept on ice until administration. Mice received an IV injection of 2.5 × 10^5^ untransduced hUC-MSCs or PBS (100 *μ*l) under anesthesia with isoflurane. Two or twenty-four hours postinjection, the animals were culled by cervical dislocation. The lungs were removed en bloc. The large airways were dissected from the peripheral lung tissue and each lung lobe was separated. The lung lobes were cut into small pieces with scissors, transferred into C-tubes (Miltenyi Biotec), and processed in digestion buffer (1 mg/ml of Collagenase D and 80 U/ml DNase I, both from Roche, in DMEM) and a GentleMACS dissociator (Miltenyi Biotec), according to the manufacturer's instructions. The lung homogenates were strained through a 70 mm nylon mesh to obtain single-cell suspensions. Red blood cells were lysed using ammonium–chloride–potassium lysis buffer (Gibco, A1049201). The resultant cells were counted using an automated cell counter (TC10, BioRad).

### 2.4. Flow Cytometry

One million cells suspended in 90 *µ*l of staining buffer (eBiosciences, 00-4222-26) were incubated with 10 *µ*l FcBlock (Miltenyi Biotec, 130-092-575) to reduce nonspecific antibody binding. The cells were stained with a mixture of fluorochrome-conjugated antibodies (see Table 1 for a list of antibodies, clones, and fluorochromes). Data were acquired on a BD CANTO II flow cytometer using BD FACSDiva software (BD Biosciences; see *Supplementary 1* which shows the instrument configuration), and compensation and data analyses were performed using the DIVA software).

To determine the proportion of innate immune cell subtypes, mice received untransduced hUC-MSCs IV and their lungs were dissociated for flow analysis. We used a range of markers to identify specific populations including granulocytes, neutrophils, monocytes, and macrophages with different specific properties (Table 2). The gating strategy followed to identify cell populations was adapted from Misharin et al. [41] and is shown in *Supplementary 2* and *3*.

### 2.5. Retrograde Perfusion Fixation

To study the cell biodistribution in the lung histologically, FLuc+ TdTomato-expressing hUC-MSCs were injected into a different cohort of mice before retrograde perfusion fixation. Two or twenty-four hours after IV administration of hUC-MSCs, the mice received an intraperitoneal overdose of pentobarbital (Pentoject, 100 *µ*l) followed by cannulation of the abdominal aorta, severing of the vena cava, and flushing of Heparin/PBS (5 IU/ml) with a manual pump at a constant pressure of 200 mbar (*Supplementary 4* shows the set-up of the perfusion pump) for 6 min to remove all blood cells, followed by 6 min perfusion with 4% w/v paraformaldehyde (PFA) to fix the whole animal. The total volume of each solution used per animal was 40 ml. The trachea was tied tightly with a surgical suture before opening the thoracic cavity for lung dissection. Finally, the lungs were postfixed in 4% PFA overnight at 4°C.

### 2.6. Immunofluorescence

Before staining, the lungs were cleared using the CUBIC protocol [48]. The cleared lungs were sucrose protected and cryo-embedded in optimal cutting temperature medium before sectioning 30 *µ*m thick sections using a cryostat (Thermo Scientific, Microm HM505E) at −20°C and stored at −80°C. All sections were washed with PBS 3x for 5 min. Tissues were incubated with primary antibodies for 2 hr at RT or O/N at 4°C, followed by PBS washes and 1 hr incubation at RT with the secondary antibodies and DAPI. After a final washing step, the sections were mounted in fluorescence mounting media (Dako, S3023). All antibodies and dilutions used can be found in Table 3.

### 2.7. Imaging

Confocal microscopy images were acquired using a Leica DMi8 with Andor Dragonfly spinning disk, coupled to an EMCCD camera using a 40x/1.3 oil objective. *Z*-stacks were captured using the 488, 561, and 637 nm laser lines. The emission filters used were 525/50, 600/50, and 700/75. Maximum intensity projections, three-dimensional reconstructions, and image analysis were done using the IMARIS (Bitplane) software package.

Cell counting with IMARIS was performed by opening *Z*-stacks in their native format, as they are automatically reconstructed into a multichannel 3D model, which eliminates the need for image preprocessing. To designate individual cells of interest, the Spots creation tool was used. In the Spots creation wizard, the source channel corresponding to the staining of interest was selected. Background subtraction was used to separate the cell from the background. The autothreshold value was utilized during background subtraction. The generated Spots were a direct map of the intensity distribution of the immunostaining of interest as detected by Imaris. Adjustments to the Spots to create an accurate representation of the staining were made using the manual spot creation/deletion tool.

### 2.8. Statistics

Data were analyzed using GraphPad Prism for Windows version 8.4.2 (GraphPad Software, Inc., San Diego, CA). Values are presented as means ± standard deviations. Comparisons between animal groups were performed using the Kruskal–Wallis test with multiple comparisons. *P* < 0.05 was considered statistically significant. The number of replicates included in the analyses is given in the figure legends.

## 3. Results

### 3.1. Effect of hUC-MSCs on the Distribution of Myeloid Cells in the Lungs

To investigate changes in biodistribution of the myeloid cells within the lungs after hUC-MSC administration, the lungs of mice that received FLuc TdTomato-expressing hUC-MSCs were fixed and used to prepare frozen sections for histology.

**Figure 1:**
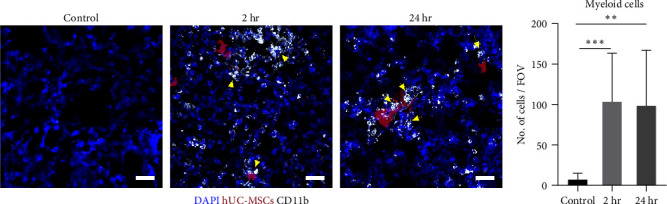
CD11b^+^ myeloid cells infiltrated into the lungs after IV administration of hUC-MSCs. (a) Representative maximum intensity projection confocal microscopy images showed CD11b^+^ cells (white) were present in the lungs of animals that received hUC-MSCs, but were not present in control animals that received saline. CD11b^+^ cells clustered around the hUC-MSCs (red) in the lungs at 2 and 24 hr after cell injection (yellow arrows). Scale bar = 30 *µ*m. (b) Immunofluorescence quantification of CD11b^+^ cells. Kruskal–Wallis test with multiple comparisons. *n* = 3,  ^*∗∗*^*P* < 0.005;  ^*∗∗∗*^*P* < 0.0005. The number of fields of view counted per condition was 27.

Using the CD11b pan-myeloid marker [49], confocal microscopy revealed that 2 hr post-IV administration of hUC-MSC, there was an increase in myeloid cells. These cells persisted in the lungs for up to 24 hr. Moreover, the cells accumulated in close proximity to the hUC-MSC clusters and fragments (Figure 1(a)), suggesting that these cells might be phagocytosing the hUC-MSCs. Quantification of myeloid cells confirmed a sharp increase in these cells at 2 hr that was sustained at 24 hr (Figure 1(b)). Therefore, immunofluorescence analysis and quantification showed that myeloid cells infiltrated the lungs after IV injection of hUC-MSCs.

### 3.2. Implementation of Gating Strategy to Identify Myeloid Cell Subtypes and Their Polarization State

We present a novel flow cytometry gating strategy to identify myeloid cells, their specific subtypes and their polarization states (*Supplementary 2* and *3*). The strategy for identifying granulocytes, polarzsed resident lung macrophages and polarized interstitial macrophages is shown in *Supplementary 2*(a); the strategy for identifying polarized infiltrating monocytes/macrophages is shown in *Supplementary 2*(b); and the strategy for neutrophils is shown in *Supplementary 3*.

### 3.3. Effect of hUC-MSCs on the Proportion, Distribution, and Polarization of Infiltrating Granulocytes within the Lung

To understand what type of myeloid cells had accumulated in the lung, we performed side-by-side flow cytometry and immunofluorescence-based analysis of cell biodistribution within the lung tissue.

**Figure 2:**
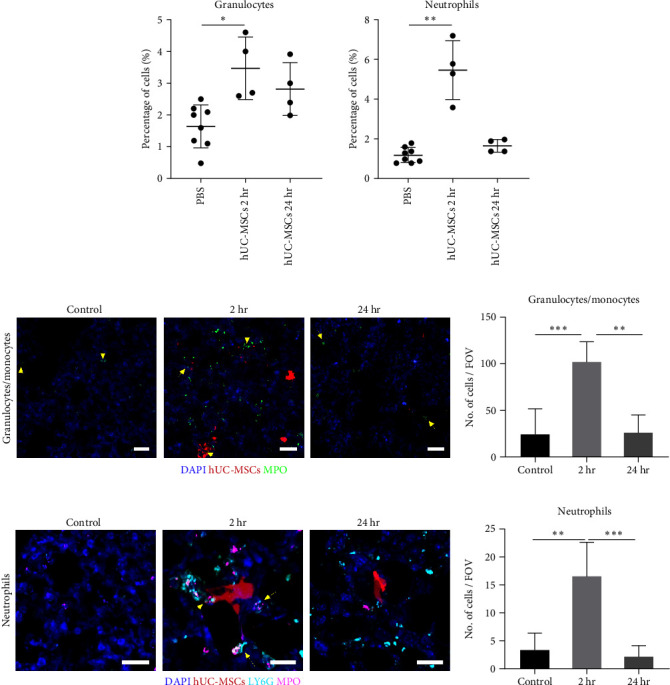
Neutrophils and other granulocytes were recruited to the lungs within 2 hr of hUC-MSC IV administration. (a) Flow cytometry showed that Cd11c^−^ CD24^hi^ granulocytes and Siglec F^−^ CD11b^hi^ CD103^−^ Ly6G^hi^ neutrophils increased 2 hr after hUC-MSC injection, and decreased at the 24 hr time point. Kruskal–Wallis test with multiple comparisons; control *n* = 8, hUC-MSC group *n* = 4,  ^*∗*^*P* < 0.05;  ^*∗∗*^*P* < 0.005. (b) MPO^+^ granulocytes/monocytes (green) clustered around the hUC-MSCs (red) in the lungs 2 hr after cell injection (yellow arrows); their levels decreased at 24 hr. Scale bar = 30 *µ*m. (c) Immunofluorescence quantification of MPO^+^ cells. Kruskal–Wallis test with multiple comparisons. *n* = 3,  ^*∗∗*^*P* < 0.005;  ^*∗∗∗*^*P* < 0.0005. (d) Ly6G + MPO (magenta + cyan) neutrophils surrounded the hUC-MSCs (red) in the lungs 2 hr after cell injection (yellow arrows). 24 hr later, neutrophil numbers had lowered but Ly6G^+^ cells were still present in the lung. (e) Immunofluorescence quantification of Ly6G^+^ MPO^+^ neutrophils. Kruskal–Wallis test with multiple comparisons. *n* = 3,  ^*∗∗*^*P* < 0.005;  ^*∗∗∗*^*P* < 0.0005. The number of fields of view counted per condition was 27.

Flow cytometric analysis revealed that within the first 2 hr of hUC-MSC administration, the proportion of granulocytes increased approximately twofold. The number of these cells decreased after 24 hr but remained 1.7x higher than in control lungs that did not receive hUC-MSCs (Figure 2(a), left).

To evaluate the tissue distribution of granulocytes and monocytes, the MPO marker was used [50, 51]. MPO-positive cells localized in the vicinity of the hUC-MSC clusters as well as in areas where cell debris was observed (Figure 2(b)), potentially indicating an active role of these cells in the clearance of the exogenously administered human cells. Quantification of the fluorescence images showed an approximately threefold increase in MPO-expressing cells at 2 hr, with a decline back to control levels at 24 hr (Figure 2(c)). The higher increase observed by histology in comparison with flow cytometry was likely due to the fact that the gating strategy excluded monocytes.

Flow cytometry demonstrated that the number of neutrophils at 2 hr had increased by approximately 4.5x, and returned to baseline after 24 hr (Figure 2(a), right). To reliably identify neutrophils by immunostaining the lung sections, we used the MPO surface marker in combination with Ly6G, which is recognized as a marker that is highly expressed by neutrophils [52]. We observed that neutrophils expressing both MPO and Ly6G localized to the vicinity of the hUC-MSCs at 2 hr. After 24 hr, cells expressing only Ly6G were observed distributed evenly throughout the lung (Figure 2(d)). In agreement with the flow cytometry data, quantification of the immunofluorescence data confirmed that these cells increased by 4x in the lungs 2 hr after cell delivery and returned to baseline levels at 24 hr (Figure 2(e)).

Together, these data showed that by 2 hr following IV administration of hUC-MSCs, granulocytes (and neutrophils in particular), accumulated in the lung, but by 24 hr, their numbers had decreased.

### 3.4. Effect of hUC-MSC on Neutrophil Extracellular Trap Formation

Given the high influx of neutrophils into the lung after IV injection of hUC-MSCs, we questioned whether NETs formed as a consequence. We stained frozen lung sections of mice that had received TdTomato-expressing hUC-MSCs for histone deacetylase 2 (HDAC2), DAPI, and MPO, which in combination are common indicators of NET formation [53].

**Figure 3:**
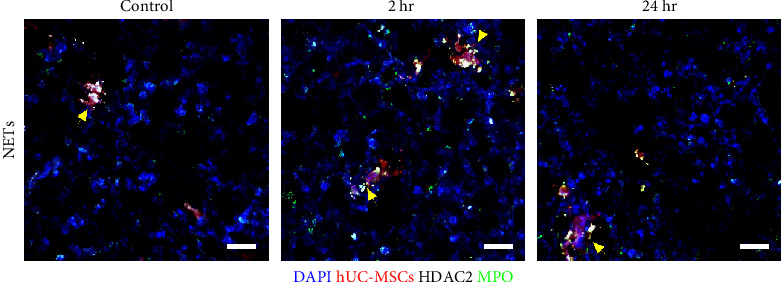
Neutrophil extracellular traps are not observed in the lungs of mice at any time point after hUC-MSC IV administration. hUC-MSCs (red), MPO (green), and HDAC II (white). Scale bar = 30 *µ*m.

Although an increase in HDAC2 was observed at both time points, neither the characteristic elongated NET structures nor colocalization with DAPI and MPO were observed (Figure 3). Thus, the NET formation was likely not induced by the administration of hUC-MSCs.

### 3.5. Effect of hUC-MSCs on the Proportion, Distribution, and Polarization of Infiltrating Monocytes and Macrophages within the Lung

Next, we determined how hUC-MSCs affected the quantity, localization, and phenotype of infiltrating macrophages and monocytes in the mouse lung. Monocytes and macrophages express similar surface molecules. The selection of markers used in our panel made it difficult to differentiate between these cell populations; therefore, they were analyzed as one population—Monocytes/M0 macrophages.

**Figure 4:**
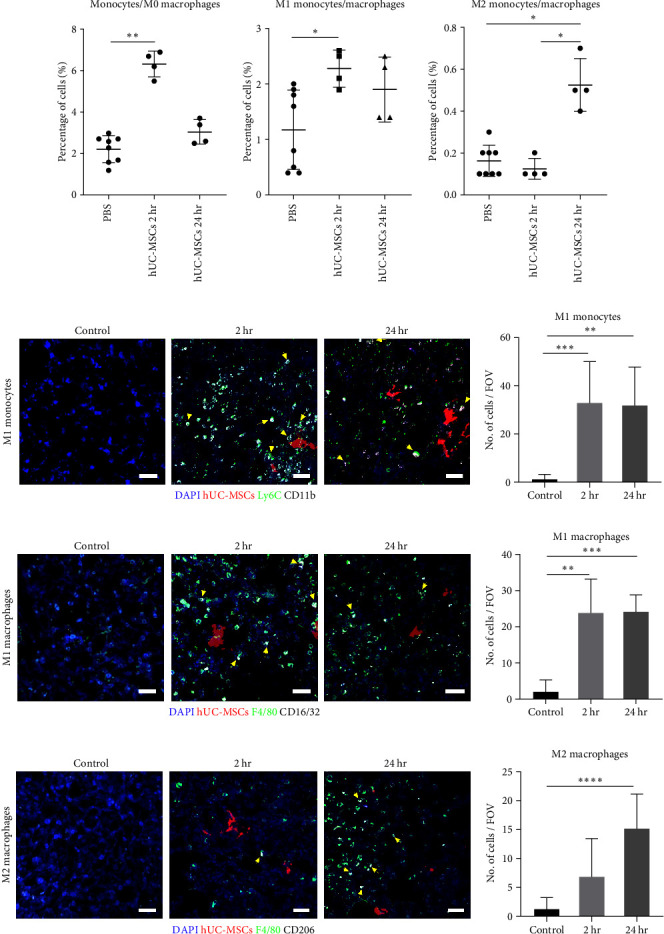
Monocytes and M0 macrophages in the lung show a two-step polarization response to IV injection of hUC-MSCs. (a) Flow cytometry showed that the CD11b^hi^ MHC II^+/−^ CD64^+/−^ monocyte and M0 macrophage populations increased at 2 hr and presented a classically activated M1 phenotype. At 24 hr there was an increase in alternatively activated M2 monocytes/macrophages. Kruskal–Wallis test with multiple comparisons. control *n* = 8, hUC-MSC group *n* = 4,  ^*∗*^*P* < 0.05. (b) Proinflammatory monocytes were immunostained for CD11b (white) and Ly6C (green). They were found evenly distributed throughout the lungs as well as close to the hUC-MSCs (yellow arrows). Scale bar = 30 *µ*m. (c) Immunofluorescence quantification of Ly6C^+^ CD11b^+^ proinflammatory monocytes. Kruskal–Wallis test with multiple comparisons. *n* = 3,  ^*∗∗*^*P* < 0.005;  ^*∗∗∗*^*P* < 0.0005. (d) Proinflammatory macrophage (F4/80 [green] + CD16/32 [white]) distribution in the lung after cell therapy (yellow arrows). Scale bar = 30 *µ*m. (e) Immunofluorescence quantification of F4/80^+^ CD16/32^+^ proinflammatory macrophages. Kruskal–Wallis test with multiple comparisons. *n* = 3,  ^*∗∗*^*P* < 0.005;  ^*∗∗∗*^*P* < 0.0005. (f) (F4/80 [green] + CD206 [white]) anti-inflammatory macrophage distribution (yellow arrowheads). Scale bar = 30 *µ*m. (g) Immunofluorescence quantification of F4/80^+^ CD206^+^ anti-inflammatory macrophages. Kruskal–Wallis test with multiple comparisons. *n* = 3,  ^*∗∗∗∗*^*P* < 0.00005. The number of fields of view counted per condition was 27.

We observed by flow cytometry that 2 hr after hUC-MSC administration, the proportion of monocytes/M0 macrophages increased by approximately 2.8x but by 24 hr, their numbers had decreased sharply (Figure 4(a), left). Proinflammatory monocytes/M0 macrophages increased approximately twofold at 2 hr and remained elevated at 24 hr (Figure 4(a), middle). On the other hand, M2 monocyte/macrophage numbers remained unchanged by 2 hr, but by 24 hr, were significantly increased by over threefold (Figure 4(a), right).

To study immune cell polarization within lung tissue sections, we performed costaining for CD11b and LY6C (proinflammatory monocytes). There was an even distribution of proinflammatory monocytes throughout the tissue without preferential accumulation around the hUC-MSCs at any time point (Figure 4(b)), suggesting that the hUC-MSCs trigger a lung-wide inflammatory response. Quantification of proinflammatory monocytes showed an infiltration of these cells into the lung at 2 hr, which was sustained at 24 hr (Figure 4(c)). Anti-inflammatory monocytes were not investigated by immunofluorescence, but it has previously been shown that there is an increase in this cell type at 24 hr postcell injection [26].

Costaining for the F4/80 and CD16/32 markers revealed that proinflammatory macrophages distributed homogeneously throughout the tissue (Figure 4(d)). Quantification showed that proinflammatory macrophages infiltrate the lung 2 hr after cell injection, with the level of these cells remaining high at 24 hr (Figure 4(e)). To identify anti-inflammatory macrophages, we used the F4/80 and CD206 markers. Double-labeled cells were observed homogeneously distributed within the tissue at 24 hr (Figure 4(f)) in agreement with the quantification which showed that anti-inflammatory macrophages increase only after 24 hr (Figure 4(e)).

Taken together, the monocyte/M0 macrophage population was significantly increased 2 hr after injection of hUC-MSCs. At this time point, an inflammatory response was observed as both monocytes and macrophages differentiated toward a proinflammatory phenotype. At 24 hr, a resolution of the inflammation phase was observed, as evidenced by the presence of increased numbers of anti-inflammatory monocytes and macrophages.

### 3.6. Effect of hUC-MSCs on the Proportion and Polarization of Lung Macrophage Subpopulations

As shown in Figure 4, IV administration of hUC-MSC increased the overall monocyte/M0 macrophage levels in the lung. Their rapid increase within a 2 hr period suggests that these cells are infiltrating from the circulation [54]. However, it is not clear whether the hUC-MSCs also have any effect on the resident macrophage populations in the lung, which comprise interstitial and alveolar macrophages. To address this, we used flow cytometry to investigate the effect of hUC-MSCs on Cd11b- CD11c^hi^ CD64^+^ alveolar and MHC II^+^ CD11b^+^ CD64^+^ CD24^−^ interstitial macrophages as well as their polarization status.

**Figure 5:**
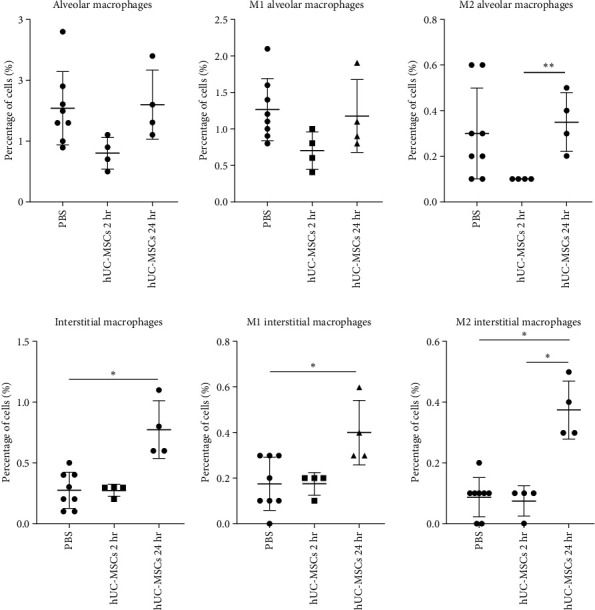
Macrophage subsets and their polarization after IV administration of hUC-MScs. (a) Flow cytometric analysis of Cd11b^−^ CD11c^hi^ CD64^+^ alveolar macrophages and their polarization in the lung. (b) MHC II^+^ CD11b^+^ CD64^+^ CD24^−^ interstitial macrophages were also analyzed for changes in their proportion and polarization by flow cytometry. Kruskal–Wallis test with multiple comparisons. control *n* = 8, hUC-MSC group *n* = 4.  ^*∗*^*P* < 0.05;  ^*∗∗*^*P* < 0.005. The number of fields of view counted per condition was 27.

The analysis showed that although not statistically significant, there were reduced numbers of alveolar macrophages at 2 hr, irrespective of subtype (Figure 5(a)). Levels of interstitial macrophages remained unchanged 2 hr postcell injection, but all subtypes analyzed had increased significantly by 24 hr (Figure 5(b)).

## 4. Discussion

**Figure 6:**
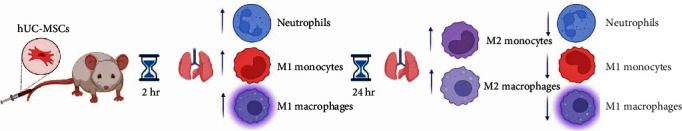
Summary of the effect of IV delivered hUC-MSCs on innate immune cells.

In this study, we observed changes in the levels of different innate immune cell subtypes after IV administration of hUC-MSCs into immunocompetent healthy mice. The key finding was that within 2 hr of hUC-MSC administration, a proinflammatory response was observed in the lung, which by 24 hr, appeared to switch to an anti-inflammatory response (Figure 6).

We injected 250,000 hUC-MSCs per animal because when injecting higher doses, we have observed elevated mortality rates. In brief, higher dosing sometimes leads to the immediate demise of the animal, which we think is due to pulmonary embolism. Using 250,000 hUC-MSCs, we have never observed any mortality. Moreover, this study demonstrated that even at a lower dosage, significant changes in the expression of cell surface markers associated with immune reactions can be observed as early as 2 hr postcell infusion.

Our findings are consistent with an earlier study showing macrophage infiltration in the lungs following IV administration of MSCs [28]. It appears that this effect on the innate immune system is not exclusive to MSCs but can occur with other cell types following IV administration and subsequent lung entrapment. For instance, the IV administration of human kidney-derived cells (hKCs) into a rat kidney injury model also led to a rapid infiltration of macrophages into the lungs, where they accumulated around the hKCs [55]. In our current study, we found that 2 hr post-hUC-MSC administration, neutrophils, and proinflammatory macrophages localized in close proximity to the MSCs, suggesting that the clearance of the exogenous cells might involve efferocytosis by phagocytic neutrophils or proinflammatory macrophages, as previously suggested [56].

After 24 hr, the levels of proinflammatory cells decreased, while an increase was observed in the levels of anti-inflammatory monocytes and macrophages. The production of suppressive myeloid cells after close interaction with MSCs has been described as one of the mechanisms by which the MSCs exert positive disease outcomes [57].

Identifying myeloid cell subtypes and their polarization state in the lung is a complex task. Several myeloid populations express similar and overlapping markers. Moreover, researchers have previously used inconsistent antibody panels, resulting in a lack of strictly defined identifiers for specific cell subsets [58]. We followed a well-established protocol [41] and modified it in accordance with our experimental goals, offering a novel approach to studying the changes in the proportion of different myeloid cells in response to hUC-MSC delivery in vivo.

We and others have shown that following IV administration, hUC-MSCs accumulate in the lungs and are cleared within 24 hr [1–3, 59]. Given that the hUC-MSCs are short-lived, their mechanisms of action in the resolution of disease are still unclear. We showed that interactions between transplanted MSCs with phagocytic myeloid cells occur shortly after administration. In agreement with our data, others have shown direct interactions of MSCs with host platelets and neutrophils in vivo [60], and that MSCs colocalize with macrophage and granulocytes ex vivo [38], suggesting that MSCs, as well as other cell types, might affect the innate immune system through cell–cell interactions in the lungs [55, 61]. Phagocytosis of exogenous MSCs by innate immune cells has been demonstrated in vivo [26, 62] and it has been found to trigger monocytes to adopt an anti-inflammatory phenotype [26]. However, a question that remains is whether innate immune cells are attracted by signals emanating from still-viable MSCs in the lung, and then play a role in inducing the death of the MSCs, or alternatively, if the MSCs start to die because the lung capillaries do not support their survival, and the innate immune cells are then attracted by signals derived from the dying MSCs.

It has been suggested that the rapid cell death of MSCs in the lungs and their subsequent efferocytosis by macrophages might be required for them to exert their therapeutic benefits [56], as shown in a mouse model of allergic asthma [63]. Moreover, clinical data from patients with graft-versus-host disease who have been administered MSCs IV, as well as data from murine models suggests that the host's cytotoxic cells actively induce the exogenous MSCs to undergo apoptosis [64]. This results in a recipient-induced immunomodulation which is required for improved outcomes [64]. In keeping with the finding that viable MSCs are not required to ameliorate injury, heat-inactivated MSCs were able to maintain their immunomodulatory capacity and reduce sepsis in mice [65, 66].

Here, we used xenogeneic cells, but studies that have used syngeneic [28] and allogeneic [67] MSCs have also observed an inflammatory immune reaction after MSC delivery, suggesting that the response is not due to the cells being from another species, but rather a response to the MSCs being present in an atypical location, which initiates a clearance mechanism [28]. In line with this, the immune system reacts to cells that are not normally in contact with the bloodstream [68]. The direct interaction between the MSCs and the blood immediately after infusion might trigger an instant blood-mediated inflammatory reaction (IBMIR), that would not be expected when administering cells that are normally present in the blood circulation, such as leukocytes [69]. IBMIR causes platelet-, coagulation-, and complement activation, and likely results in the MSCs being destroyed quickly, inducing the innate immune system to eliminate them [70].

Regarding lung resident macrophages, our data showed that the hUC-MSCs did not induce changes in the proportion or phenotype of alveolar macrophages. In contrast, others have shown that MSCs induce a slight increase in alveolar macrophages following IV administration [63]. Moreover, alveolar macrophages were observed to efferocytose the exogenous MSCs, which polarized them towards an anti-inflammatory phenotype [63, 71, 72]. This might be explained by the fact that healthy mice were used in the current study, while the three studies cited above used a mouse model of allergic asthma. Alveolar macrophages are the first immune effector cells at the air–lung interphase [73], meaning that the induction of asthma might have activated, primed, and mobilized the alveolar macrophages before MSC therapy [74]. Thus, it is difficult to attribute the observed effects solely to the delivery of MSCs.

As discussed above, the disease context can influence MSC behavior [75, 76]. MSCs can either promote or suppress the immune response as shown by in vitro culture of MSCs exposed to different clinical bronchoalveolar lavage samples representing a wide range of lung pathologies [76]. Regarding lung interstitial macrophages, we observed a 2.8-fold increase in interstitial macrophages at 24 hr, which agrees with Pang et al. [63] who observed that lung interstitial macrophages play an important role in the clearance of exogenous MSCs.

How the immunoregulatory properties of MSCs relate to their beneficial effects in disease and injury models remains unclear. Nevertheless, immunomodulation by innate immune cells mediated by the MSC secretome as well as by direct interaction with viable, apoptotic, inactivated, and fragmented MSCs has been established [56]. Importantly, MSCs appear to be able to induce therapeutic effects without long-term engraftment [77].

A possible limitation of our study is that we only used MSCs from a single umbilical cord. However, we have recently compared the properties of hUC-MSCs from three different donors and found that their properties are similar, in regard to proliferation rate and surface marker expression, and their survival following intravenous administration in mice [78].

## 5. Conclusions

We performed a comprehensive flow cytometry and histological analysis of mouse lungs following IV administration of hUC-MSCs to investigate the fate of the hUC-MSCs and their effect on the cells of the innate immune system. We showed that in healthy, immunocompetent mice, an inflammatory response occurred in the lungs 2 hr after cell delivery. This response was dominated by an increase in granulocytes—particularly neutrophils—and proinflammatory monocytes and macrophages. These innate immune cells were frequently observed in proximity to the hUC-MSCs, which may indicate that they participate in their clearance by means of phagocytosis. After 24 hr, a resolution of the inflammatory phase was observed as anti-inflammatory monocytes and macrophages became more prevalent in the lung. These processes might be involved in the immunomodulatory response following MSC infusion in models of disease. Further research is necessary to ascertain the exact cause of the immune response to better tailor cell therapies to specific conditions.

## Figures and Tables

**Table 1:** Antibodies used for flow cytometry.

Conjugated antibody	Host/isotype	Clone	Dilution	Catalog no.
CD45 FITC	rat IgG2b*κ*	30F11	1 : 50	130-116-500
CD11b VIOBLUE	Rat IgG2b, k	M1/70.15.11.5	1 : 50	130-113-238
Cd11c APC-Vio770	Hamster IgG	N418	1 : 50	130-122-016
CD64 APC	Human IgG1	REA286	1 : 50	130-126-950
CD24 PE-Vio770	rat IgG2b*κ*	M1/69	1 : 10	130-102-736
MHCII PE	rat IgG2b*κ*	M5/114.15.2	1 : 10	130-102-186
CD71 PERCPVIO700	Human IgG1	REA627	1 : 50	130-128-620
Siglec-F PE-Vio770	Human IgG1	REA798	1 : 50	130-112-334
CD103	Hamster IgG	2E7	1 : 50	130-121-442
Ly6G	Human igG1	REA526	1 : 50	130-120-803

*Note*. All antibodies were purchased from Miltenyi Biotec.

**Table 2:** Surface markers used to identify immune cells by flow cytometry and immunofluorescence.

Cell type	Flow cytometry	Immunofluorescence	Reference
Granulocytes	Cd11c^−^ CD24^hi^	Myeloperoxidase (MPO)	[41]
Neutrophils	Siglec F^−^ CD11b^hi^ CD103^−^ Ly6G^hi^	MPO + Ly6G	[41]
Monocyte/M0 macrophages	CD11b^hi^ MHC II^+/−^ CD64^+/−^	N/A	[41]
Alveolar macrophages	Cd11b^−^ CD11c^hi^ CD64^+^	N/A	[33]
Interstitial macrophages	MHC II^+^ CD11b^+^ CD64^+^ CD24^−^	N/A	[42]
Proinflammatory monocytes	CD11b^hi^ MHC II^+/−^ CD64^hi^ CD71^−^	Cd11b + Ly6C	[43]
Anti-inflammatory monocytes	CD11b^hi^ MHC II^+/−^ CD71^hi^	N/A	[44]
Proinflammatory macrophages	CD11c^hi^ CD11b^+/−^ CD24^−^ CD64^hi^ CD71^−^	F4/80 + Cd16/32	[45, 46]
Anti-inflammatory macrophages	CD11c^hi^ CD11b^+/−^ CD24^−^ CD64^−^ CD71^hi^	F4/80 + CD206	[47]

**Table 3:** Antibodies used for immunofluorescence.

	Host	Clone	Isotype	Dilution	*T*°/time	Manufacturer
F4/80 FITC	Human	REA126	IgG1	1 : 50	RT/2 hr	Miltenyi biotech (130-117-509)
CD16/32	Human	REA377	IgG1	1 : 10	RT/2 hr	Miltenyi biotech (130-107-066)
Ly6C	Rat	HK1.4	IgG2c, k	1 : 200	4/ON	Biolegend (128001)
MPO	Goat	Polyclonal	IgG	1 : 100	4/ON	R&D Systems (AF3667-SP)
HDAC2	Rabbit	Polyclonal	IgG	1 : 250	RT/2 hr	Sigma–Aldrich (HPA011727)
CD11b APC	Rat	M1/70.15.11.5	IgG2b, k	1 : 50	RT/2 hr	Miltenyi biotech (130-113-231)
Ly6G APC	Human	REA526	IgG1	1 : 50	RT/2 hr	Miltenyi biotech (130-120-803)
CD163	Rabbit	Polyclonal	IgG	1 : 100	RT/2 hr	Invitrogen (PA5-78961)
Alexa Fluor® 750 donkey antirat	Donkey	Polyclonal	IgG	1 : 200	RT/1 hr	Abcam (ab175750)
Alexa Fluor® 647 goat antirabbit	Goat	Polyclonal	IgG	1 : 1,000	RT/1 hr	Invitrogen (A-212465)
Alexa Fluor® 647 goat antihamster	Goat	Polyclonal	IgG	1 : 1,000	RT/1 hr	Invitrogen (A-21451)
Alexa Fluor® 633 goat antirat	Goat	Polyclonal	IgG	1 : 1,000	RT/1 hr	Invitrogen (A-21094)

## Data Availability

The data that support the findings of this study are available to download from Zenodo at https://doi.org/10.5281/zenodo.7113094.

## Supplementary Materials

Supplementary 1 Flow cytometer configuration.

Supplementary 2 Gating strategy used to identify immune-cell subsets in the mouse lung after IV administration of hUC-MSCs or saline.

Supplementary 3 Gating strategy used to identify neutrophils in the mouse lung after IV injection of hUC-MSCs or saline.

Supplementary 4 Perfusion pump set up.
