# Supplementary figures and images for: Intravenous Administration of Human Umbilical Cord Mesenchymal Stromal Cells Leads to an Inflammatory Response in the Lung
Source: Stem Cells Int. 2023 Sep 5;2023:7397819. doi: 10.1155/2023/7397819 (PMC10497368; doi:10.1155/2023/7397819)

Supplementary Figures


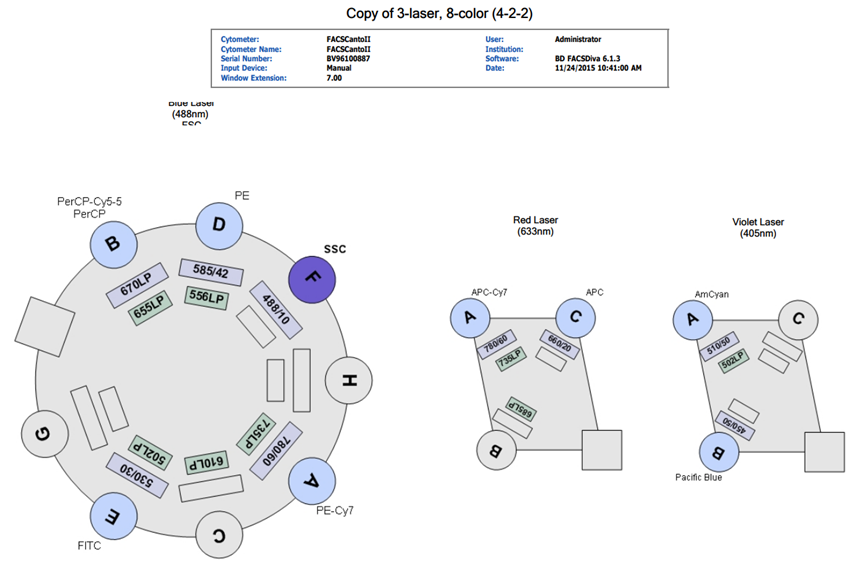


*Supplementary figure 1.9BD FACS CANTO II configuration.*

Supplement: Supplementary 1 — Flow cytometer configuration. [file 7397819.f1.docx]
