# Supplementary material for: Intravenous Administration of Human Umbilical Cord Mesenchymal Stromal Cells Leads to an Inflammatory Response in the Lung
Source: Stem Cells Int. 2023 Sep 5;2023:7397819. doi: 10.1155/2023/7397819 (PMC10497368; doi:10.1155/2023/7397819)
Supplement: Supplementary 2 — Gating strategy used to identify immune-cell subsets in the mouse lung after IV administration of hUC-MSCs or saline. [file 7397819.f2.docx]

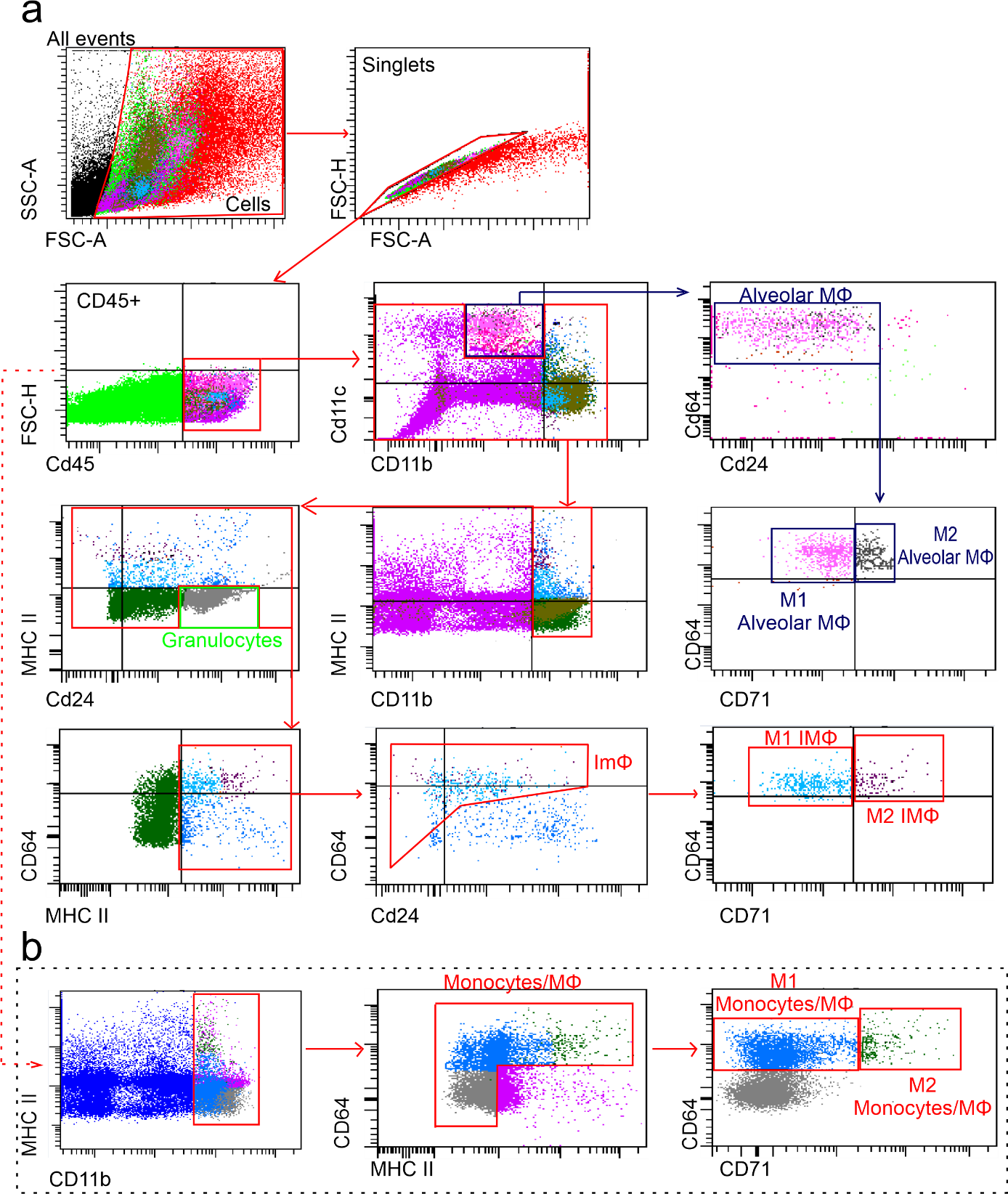


*Supplementary figure 2. Gating strategy used to identify immune-cell subsets in the mouse lung after IV administration of hUC-MSCs or saline. After enzymatic and mechanical digestion of mouse lungs, debris and doublets were excluded. Leukocytes were identified by CD45 staining. a) A sequential gating strategy was used to identify alveolar macrophages (MΦ) (CD11b- CD11chi), granulocytes (CD11c- CD24hi), interstitial macrophages (CD11b+ MHC II+ CD64+ CD24−). To identify classically- (M1) and alternatively activated (M2) cell types, CD64 (pro-inflammatory marker) and CD71 (anti-inflammatory marker) were used. B) A parallel gating strategy was used to identify monocytes/M0 MΦ (CD11bhi MHC II+/- CD64+/−). The polarization status toward a pro- or anti-inflammatory phenotype was assessed by the expression of CD64 and CD71, respectively. Data acquired in a BD CANTO II flow cytometer, the configuration of which can be seen in Supplementary Figure 1.*
