# Supplementary material for: Intravenous Administration of Human Umbilical Cord Mesenchymal Stromal Cells Leads to an Inflammatory Response in the Lung
Source: Stem Cells Int. 2023 Sep 5;2023:7397819. doi: 10.1155/2023/7397819 (PMC10497368; doi:10.1155/2023/7397819)
Supplement: Supplementary 3 — Gating strategy used to identify neutrophils in the mouse lung after IV injection of hUC-MSCs or saline. [file 7397819.f3.docx]

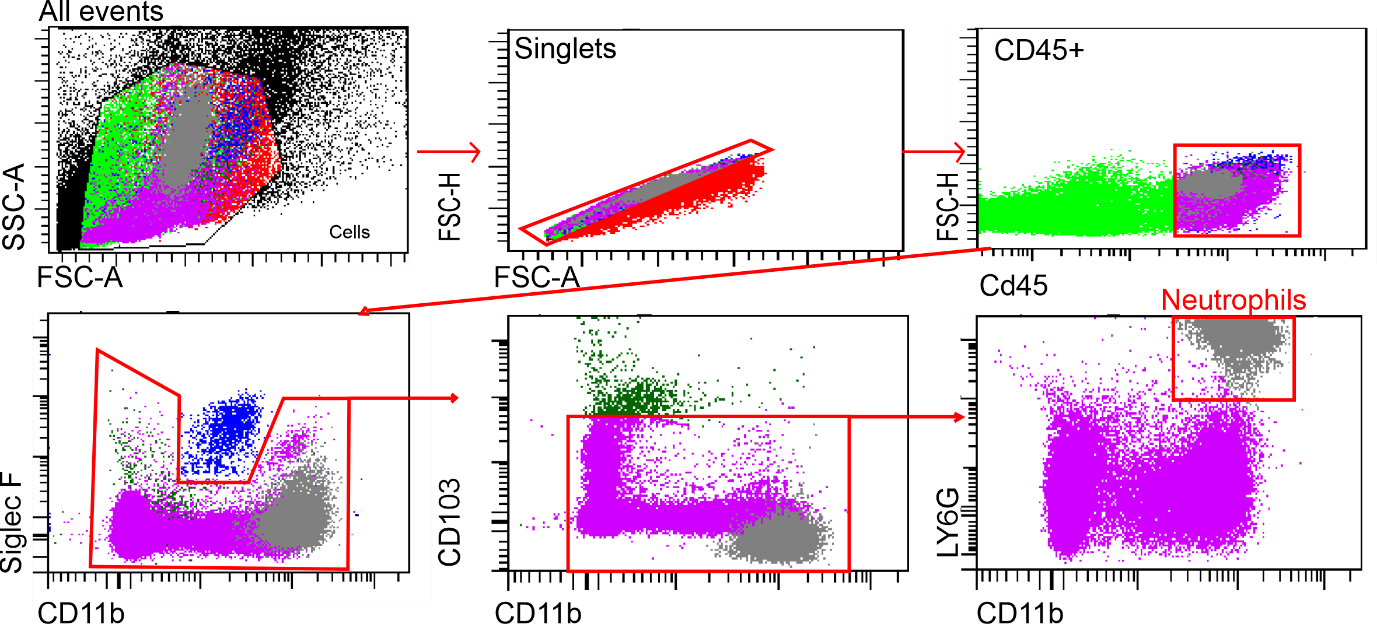


*Supplementary figure 3. Gating strategy used to identify neutrophils in the mouse lung after IV injection of hUC-MSCs or saline. Lung enzymatic and mechanic digestion was performed. Debris and doublets were excluded. CD45 staining was used to identify leukocytes. Neutrophils were gated as Siglec F^-^ CD11b^hi^ CD103^-^ Ly6G^hi^.*
